# Predictors of Patient Satisfaction with Nursing Care in an Orthopedic and Urologic Population

**DOI:** 10.22086/gmj.v0i0.1305

**Published:** 2018-12-31

**Authors:** Hamidreza Sadeghi-Gandomani, Negin Masoudi Alavi, Mohammad Afshar

**Affiliations:** ^1^Kashan University of Medical Sciences, Kashan, Iran; ^2^Trauma Nursing Research Center, Kashan University of Medical Sciences, Kashan, Iran; ^3^Trauma Nursing Research Center, Kashan University of Medical Science, Kashan, Iran; ^4^Department of Operating Room, Faculty of Nursing and Midwifery, Kashan University of Medical Sciences, Kashan, Iran

**Keywords:** Patient Satisfaction, Nursing Care, Orthopedic Trauma, Urologic Disease, Surgical Ward

## Abstract

**Background:**

Patient satisfaction is a multi-dimensional concept that can be influenced by social, cultural, and economic factors. This study was designed to evaluate the determinants that could predict patient satisfaction with nursing care in an orthopedic and urologic Population at a selected surgical ward in Kashan, Iran during 2018.

**Materials and Methods:**

This cross-sectional study was performed on 250 male patients with orthopedic and urologic diagnosis that were hospitalized in men surgical ward of Shahid Beheshti hospital affiliated to Kashan University of Medical Sciences. Patients satisfaction was assessed by a researcher-made questionnaire. The data was analyzed by the independent t-test, analysis of variance, correlation, and multiple regression analysis statistical tests.

**Result:**

The mean score of patient satisfaction was 136.05±48.10 (possible range 45-225). The age, marital status, education, diagnosis, the length of stay in the hospital, and the verbal communication of nurses with patients showed a significant correlation with patient satisfaction (P<0.001). Regression analysis revealed that age (P=0.013), marital status (P<0.001), education level (P=0.038), the length of stay in hospital (P=0.002), and verbal communication (P<0.001) could make a meaningful model with patient satisfaction in surgical wards.

**Conclusion:**

Many personal and professional factors can determine patient satisfaction in orthopedic and urologic population. Verbal communication between nurses and patients is an important factor for patient satisfaction.

## Introduction

Injuries due to different types of trauma are one of the main causes of mortality and disability worldwide [1]. The World Health Organization (WHO) consid­ered trauma as an important issue that merits more atten­tion [2]. Approxi­mately, five million people die annually (570 individuals per hour) in accidents that can be prevented [3]. Ortho­pedic injuries, including soft tissue, muscle, or bone in­juries, are the most frequent injuries in traffic and other types of accidents [1].

Nowadays, orthopedic trau­mas and urologic diseases requiring surgery are markedly increased and cause critical individual and social disabilities, loss of work days, and less social activities [4].

Despite abun­dant epidemiologic data on the nature of such injuries, few studies are focusing on patient satisfaction in this population [5].

Patient satisfaction with nursing care is one of the key determinants of overall satisfaction of patients and quality evaluations in hospitals, especially in patients with trauma [6].

Nursing care is the main experience that patients encounter during their stay in hospitals, and it constitutes a crucial part of overall satisfaction [7].

Patient satisfaction is a multi-dimensional concept that can be influenced by social, cultural, and economic factors [8,9]. Risser conceptualized patient satisfaction as the degree of congruency between what the patient expects and what is offered by the nursing care [10].

The main goal of health systems is to provide high-quality services, and patient satisfaction is a crucial indicator of this quality. When patients feel that their expectations are met, their compliance to treatment increases and this can improve their physical and mental healing process [11-13]. Conversely, if their expectations are not met, they feel anxious and stressed and cannot respond well to interventions. It delays recovery, prolongs hospitalization, and increases costs of treatment [14]. Therefore, patient satisfaction is one of the prerequisites for every therapeutic procedure [11].

Today, measuring patient satisfaction is an integral part of hospital/clinic management strategies across the globe. Also, the quality assurance and accreditation process in most countries requires that the measurement of patient satisfaction on a regular basis [15]. An important step towards improving the quality of care and ensuring that local health services meet patients’ needs is to ask patients what they think about the services they received [16]. It is a fact that satisfaction influences whether a person seeks medical advice complies with treatment, and maintains a sustained relationship with practitioners [15,17].

Studies show that giving information to the patient about his/her improvement, the skill and knowledge of nurses in patient education, the waiting time to visit physician, and the proper relationship between patient and health personnel are determinants of patient satisfaction [13,18].

Studies indicate that patient satisfaction can be influenced by many factors, both endogenous and exogenous to the care received. Patient characteristics such as gender, marital status, education level, and health condition are associated with satisfaction, although they may not be as important as factors specific to the care setting [18].

Despite the large number of studies addressing this topic, the results are still inconclusive and contradictory and still many dimensions of this concept are not known well [19-21].

A study in Turkey showed that patients in the age group of 40 to 59 years with low education reported the highest satisfaction [22]. Another study in Ethiopia indicated that the highest satisfaction could be observed in patients within the age range of 18-30 years [23]. Mensa *et al*. found that patients with an academic degree had 1.6 times greater satisfaction compared with the ones with less education [7].

In a study in the United States, satisfaction was higher in married patients [24], while another study revealed higher satisfaction in single patients [25].

It seems that there are still many questions about the predictors of patient satisfaction and this concept needs further investigations [19-21]. In Iran, there is also a need for further studies using culturally valid instruments. Patients with traumatic injuries and urologic diseases are especially important in this regard, since such patients receive more invasive treatments and encounter very difficult moments during their stay in hospital that make nursing care more important and vital [22,23].

The current study had a significant input to assess the level of clients’ satisfaction, identify the factors affecting patients’ satisfaction, and provide suggestions for improving health service delivery that helps to fill research knowledge gaps, which ultimately contributes to the enhancement of quality of patient care in hospitals and improves the level of clients’ satisfaction. The current study was designed to evaluate the predictors of patient satisfaction in orthopedic and urologic population at a selected surgical ward of Shahid Beheshti Hospital in Kashan, Iran in 2018.

## Materials and Methods

### Study Design and Subjects

The current cross-sectional study was performed on sequential patients referred to the males` surgical ward at Shahid Beheshti hospital from November 2017 to February 2018. This ward has 36 beds and provides services to male patients undergoing orthopedic and urologic surgeries. Shahid Beheshti hospital is the only general hospital in Kashan, a city in the center of Iran, with 400 beds that provides services to a population of 300,000 [26].

### Inclusion and Exclusion Criteria

The sequential and convenience sampling methods were used in the current study. The inclusion criteria were staying in the ward for at least 24 hours, undergoing surgery, age 18 years or above, interested to participate in the study and signing the consent form, no history of diagnosed mental disorders, and full orientation. Patients with severe pain or other physical problems that could not complete the questionnaire were excluded from the study.

### Sample Size Calculation

The sample size was determined by single population proportion formula (n = [(Zα/2)^2^ × P (1-P)]/D^2^), by assumption, a patient satisfaction of 78% [2], 95% confidence interval =1.96, the margin of error 5%. The sample size was calculated 264 and considering 10% possible dropouts; the total sample size was considered 290 patients.

### Data Collection

The questionnaire had two parts. In the first part, demographic characteristics such as gender, age, marital status, and education were recorded. The variables related to hospitalization such as diagnosis, length of hospital stay, and the kind of surgery were also recorded. In the second part, the patient satisfaction questionnaire developed by Ghods *et al*., [27] was used. This questionnaire includes 45 items and evaluates patient satisfaction in the four dimensions of empathy in caring, proficiency, negligence, and cleanliness. The items are scored based on a five-point Likert scale (i.e., 1=completely disagree, 2=disagree, 3= no idea, 4=agree, and 5= completely agree). The maximum and minimum possible scores are 45 to 225, respectively, with higher scores indicating higher patient satisfaction. According to the cutoff points proposed by Ghods *et al*., scores can be categorized as follows: 45-89: dissatisfaction, 90-134: low satisfaction, 135-179: intermediate satisfaction, and 180-225: high satisfaction. The questionnaire had acceptable validity and internal consistency (0.7). According to the current study, the test-retest reliability of 25 patients ten days after discharge was 0.96 [27].

The patients completed the questionnaire on the last day of their hospital stay. The questionnaire was given to the patients, and they were asked to complete it in the hospital ward. If they had difficulty in reading the items, it was completed through the interview.

### Statistical Analysis

The data was analyzed by SPSS-16 (SPSS Inc., Chicago, IL, USA). To describe the patient satisfaction, the mean, and standard deviation, and frequencies were used. The normality of the data was analyzed with Kolmogorov Smirnov test. The relations between patient satisfaction and other variables such as age and education were analyzed with the independent t-test, analysis of variance, and Pearson or Spearman correlations. The backward multiple regression analysis was used to analyze the variables that could predict the patients’ satisfaction as the dependent factor.

## Result

Overall, 290 questionnaires were distributed, of which 270 were completed; however, 20 returned questionnaires were excluded due to unanswered items, and finally, 250 (86% response rate) questionnaires were analyzed. The results showed that 133 (53.4%) patients were single, and in terms of education level, 45 (18%) patients were illiterate, 23 (9.2 %) had elementary education, 66 (26.4%) were under diploma, 52 (20.8%) had high school diploma, and 64 (25.6%) patients had a university degree. The diagnosis of 110 (44%) patients was double fracture of the tibia and fibula, 42 (16.6%) had double fracture of the forearm, 39 (15.7%) patients had fracture of the hip or femur, 31 (12.4%) men underwent prostate surgery and 28 (11.3%) men had varicoceles . The majority of the patients (n=117; 46.8%) stayed one or two days in the ward, 107 (42.8%) patients stayed three to four days, and 26 (10.4%) patients stayed five days or more in the ward.

The mean score of patient satisfaction was 136.05±48.10. About half of the patients (n= 130; 52%) reported that nurses had respectful relationship with them, and 127 (50.8%) patients stated that nurses provided adequate information about the treatment schedule, reasons of physician’s delay, the surgical procedure, administered drugs, laboratory tests, and care after discharge.

Pearson’s correlation coefficient showed a significant and positive correlation between patient satisfaction score and age.

There was a significant relationship between marital status, education, diagnosis, length of hospital stay, and verbal communication of nurses with patient satisfaction (Tables-1 and 2). Patients with less education and shorter length of stay in the ward had higher satisfaction (Table-1). The variables that were significantly associated with patient satisfaction were entered into multiple regression analysis, and backward method was used. Age, education, marital status, length of hospital stay, and verbal communication with nurses made a reliable model that could explain 57% of patient satisfaction (Table-3).

**Table 1:** The Correlation of Patient Satisfaction with Some Basic Variable in Men Surgery Ward

**Variables**	**Age**	**Education**	**The length of stay in the hospital**
Patient satisfaction of nursing care	0.146	-0.59	-0.73

**Table 2:** The Relation Between Patient Satisfaction and Individual and Social Variables in Surgical Ward

**Variables**		**N (%)**	**Patient satisfaction** **mean±SD**	**P-value**
**Marital status**	Single	133(53.4)	135.14±24.82	<0.001
	Married	117(46.6)	137.16±27.68	
**Education level**	Illiterate	45(18)	140.36±27.96	<0.001
	Primary	23(9.2)	137.90±29.82	
	Under diploma	66(26.4)	134.65±27.47	
	Diploma	52(20.8)	133.73±26.95	
	Academic	64(25.6)	133.34±28.97	
**Diagnosis**	Fracture in leg	110(44)	136.83±34.71	<0.001
	Fracture in hand	42(16.6)	136.02±36.59	
	Fracture in femur	39(15.7)	134.40±31.71	
	Prostate Surgery	31(12.4)	135.04±35.54	
	Varicocelectomy	28(11.3)	138.68±33.45	
**Length of stay in the hospital**	1-2 days	117(46.8)	139.70±33.81	<0.001
	3-4 days	107(42.8)	133.68±31.21	
	More than 5 days	26(10.4)	131.38±32.19	
**Communication with the patient**	Yes	130(52)	139.62±36.06	<0.001
	No	120(48)	132.64±31.08	
**Explaining the reason for physician delay**	Yes	127(50.8)	139.67±36.24	<0.001
	No	123(49.2)	132.76±31.87	
**Describing the treatment process**	Yes	127(50.8)	139.70±36.07	<0.001
	No	123(49.2)	132.72±31.25	

**Table 3:** Multiple Regression Analysis of Patient Satisfaction as the Dependent Variable in Surgical Ward

**Dependent variable**	**Independent variable**	β*	**Std.Error** ^†^	**Beta**	**t**	**P-value**
**Patient satisfaction**	Constant	129.299	1.895	-	68.236	<0.001
	Marital status	2.033	0.528	0.208	3.851	<0.001
	Education	-0.416	0.199	-0.120	-2.088	0.038
	Length of stay	-1.378	0.443	-0.188	-3.113	0.002
	Communication	4.381	0.570	0.451	7.692	<0.001
	Age	0.055	0.022	0.156	2.503	0.013
	R=0.759		R^2^=0.756	Adjusted R^2^=0.570		

*Regression coefficient; ^†^Standard error

In the current study, patient satisfaction with nursing care score was 136.05 indicating a moderate satisfaction. Many other studies showed various levels of patient satisfaction. A study in Tanzania reported that patient satisfaction was low [28], and another study in Iran also found an intermediate satisfaction level with nursing care [29]. Some other studies in Korea and Brazil reflected high satisfaction [18,30-32]. There was another study in Iran that showed a high level of satisfaction with nursing care [33]. These findings indicate that patient satisfaction can vary according to the setting of the study and sociocultural factors. Today, patients are more aware of their rights and have more expectations. This can be a challenge when the basic problems in the nursing system are considered; problems such as nursing shortage and deep dissatisfaction of nurses with their working conditions and salaries. It is crucial to study the reasons for patients’ dissatisfaction and provide a better caring environment.

There are also some concerns regarding the factors affecting patient satisfaction in different studies. For example, the setting of the study is very important. The current study was performed in a male` surgical ward; therefore, results might not be similar to those of other wards.

The instruments used for data collection might also evaluate just some limited aspects of nursing care since this concept has different dimensions and the instrument should be carefully validated or designed according to social and cultural differences.

Although social and cultural factors can influence patient satisfaction with nursing care, some concepts are important in any country. Respect and efficient communication are the concepts that can serve crucial roles in patient satisfaction in any hospital in the world.

The current study, a study in Ireland [34], and another study in the USA [35] also found that efficient and respectful professional relationship of nurses with patients was the strongest predictor for patient satisfaction.

In South Africa, the nurse-patient relationship was the main determinant of quality of care and patient satisfaction [36]. Some other studies also showed the importance of the nurse-patient relationship and interpersonal communication skills in patient satisfaction [37-40]. Such skills help nurses to gather information more efficiently, provide counseling and education, help physician for better diagnosis, and respond to patients’ requests, and complaints more effectively [41]. Improving nurses’ interpersonal communication skills should be one of the goals of policymakers.

Marital status was one of the predictors of patient satisfaction. Married males were more satisfied with nursing care. Xiao and Barber [42], and Woodside *et al*. [43] also reported the same result. Another study in the United States also found that married males were more satisfied with nursing care [24]. This might be related to the more realistic expectations of married males compared to the single ones, or to the support of their wives during hospitalization that ask for better care for their loved ones.

Education was another predictor. Patients with lower level of education reported higher satisfaction score. Dorigan at al. also found that patients with lower education had higher satisfaction with nursing care [18]. Two studies conducted in the United Arab Emirates [44] and Saudi Arabia [37] reported the lowest satisfaction in patients with university degrees. Many other studies in Africa, Iran, and other countries also reported the same result [25,45-48]. This might be related to the greater expectations of educated individuals and the fact that in hospitals and health centers most of the time people are treated equally without considering their education. Also, highly educated persons might have better access to information and ask for better care and they can better perceive the shortage of services.

The length of stay was another predictor, that is, patients that were discharged from hospital sooner were more satisfied. Mensa *et al*. also found that patient satisfaction was higher in patients that stayed in the hospital for one to five days compared with the ones that stayed in hospital more than 15 days [7]. Some other studies also reported the inverse relationship between the length of hospital stay and patient satisfaction [25,35,49,50]. Reduction of the length of hospital stay should be an objective in every health center. This can also have economic benefits for patients and health systems.

Patients’ age was a predictor for their satisfaction; older patients reported higher satisfaction. Fan also found higher satisfaction in older patients [51]. A study on 39 hospitals in Germany also found higher satisfaction with nursing care in older adults [17]. Most studies confirmed this finding [13,52,53]. One possible reason regarding the higher satisfaction of older participants could be that older patients may be treated differently, that is, more gently than younger ones [54].

Patient satisfaction is not a clearly defined concept, although it is identified as an essential quality outcome indicator to measure the success of service delivery systems. Patients’ evaluation of care is important to provide the improvement opportunity such as strategic framing of health plans, which sometimes exceed patient expectations and benchmarking. The advantages of the current study rely heavily on using standardized, psychometrically tested data collection approaches.

The current study had some limitations including performing the survey only on males admitted to surgical wards, which limits the generalizability of the results, and not being able to represent causal relationships due to the cross-sectional design of the study. One of the strengths of the study was the instrument used for data collection, which was a culturally designed and valid questionnaire. The findings of the current study can help clinicians and nursing managers to better understand patient satisfaction and its different aspects.

## Conclusion

Patient satisfaction in males hospitalized in a surgical ward was intermediate. The concept of patient satisfaction is multidimensional, and many individual and social factors such as age, marital status, education, and period of stay can influence it. Efficient verbal communication of nurses with patients is the most modifiable factor in patient satisfaction. Patient satisfaction is the main component of quality care in surgical wards; accordingly, any interventions to improve this indicator are of great importance. Holding interpersonal communication skills workshops for nurses and encouraging nurses that have good communication with patients are suggested. Further interventional studies in this area are also recommended.

## Conflict of Interest

There is no conflict of interest.

## References

[1] Soleymanha M, Mobayen M, Asadi K, Adeli A, Haghparast-Ghadim-Limudahi Z (2014). Survey of 2582 cases of acute orthopedic trauma. Trauma Mon.

[2] Toroyan T, Peden MM, Iaych K (2013). WHO launches second global status report on road safety. Inj Prev.

[3] Celso B, Tepas J, Langland-Orban B, Pracht E, Papa L, Lottenberg L (2006). A systematic review and meta-analysis comparing outcome of severely injured patients treated in trauma centers following the establishment of trauma systems. J Trauma Acute Care Surg.

[4] Urquhart DM, Edwards ER, Graves SE, Williamson OD, McNeil JJ, Kossmann T (2006). Victorian Orthopaedic Trauma Outcomes Registry (VOTOR) Project Group.
Characterisation of orthopedic trauma admitted to adult level 1 trauma centres. Injury.

[5] Morris BJ, Richards JE, Archer KR, Lasater M, Rabalais D, Sethi MK (2014). Improving patient satisfaction in the orthopedic trauma population. Orthop Trauma.

[6] Messina G, Vencia F, Mecheroni S, Dionisi S, Baragatti L, Nante N (2015). Factors affecting patient satisfaction with emergency department care: an Italian rural hospital. Glob J Health Sci.

[7] Mensa M, Taye A, Katene S, Abera F, Ochare O (2017). Determinants of Patient Satisfaction Towards Inpatient Nursing Services and its Associated Factors in, Gamo Gofa Zone, SNNPR, Ethiopia. MOJ Clin Med Case Rep.

[8] Lee DS, Tu JV, Chong A, Alter DA (2008). Patient satisfaction and its relationship with quality and outcomes of care after acute myocardial infarction. Circ.

[9] Klotz T, Zumbe J, Velmans R, Engelmann U (1996). The determination of patient satisfaction as a part of quality management in the hospital. Dtsch Med Wochensch.

[10] Risser NL (1975). Development of an instrument to measure patient satisfaction with nurses and nursing care in primary care settings. Nurs Res.

[11] Strasen L (1988). Incorporating patient satisfaction standards into quality of care measures. J Nurs Admin.

[12] Mack JL, File KM, Horwitz JE, Prince RA (1995). The effect of urgency on patient satisfaction and future emergency department choice. Health Care Manage Rev.

[13] Batbaatar E, Dorjdagva J, Luvsannyam A, Savino MM, Amenta P (2017). Determinants of patient satisfaction: a systematic review. Perspect Public Health.

[14] Özsoy S, Özgür G, Durmaz Akyol A (2007). Patient expectation and satisfaction with nursing care in Turkey: a literature review. Int Nurs Rev.

[15] Assefa F, Mosse A (2011). Assessment of clients’ satisfaction with health service deliveries at Jimma University specialized hospital. Ethiop J Health Sci.

[16] Westaway MS, Rheeder P, Van Zyl DG, Seager JR (ug 1). Interpersonal and organizational dimensions of patient satisfaction: the moderating effects of health status. Int J Qual Health Care2003 A.

[17] Fitzpatrick R. Surveys of patient satisfaction: important general considerations. MBJ 1991; 302: 887-9. Rev Bras Epidemiol. 10.1136/bmj.302.6781.887 PMC1669267 1821624

[18] Dorigan GH, Oliveira HC, Guirardello EdB (2015). Predictors Of Patients’experiences And Satisfaction With Nursing Care In Medical-Surgical Wards. Texto & Contexto-Enfermagem.

[19] Jackson JL, Chamberlin J, Kroenke K (2001). Predictors of patient satisfaction. Soc Sci & Med.

[20] Footman K, Roberts B, Mills A, Richardson E, McKee M (2013). Public satisfaction as a measure of health system performance: a study of nine countries in the former Soviet Union. Health Policy.

[21] Alberto Sánchez C, Javier Prado-Galbarro F, García-Pérez S, Sarría Santamera A (2014). Factors associated with patient satisfaction with primary care in Europe: results from the EUprimecare project. Qual Prim Care.

[22] DaÄŸdeviren N, Akturk Z (2004). An evaluation of patient satisfaction in Turkey with the EUROPEP instrument. Yonsei Med J.

[23] Mulugeta M, Berhe A, Shumye A, Yohannes A (2014). Assessment of adult patients’ satisfaction and associated factors with nursing care at Tikur Anbassa hospital. Int J Nurs Midwifery.

[24] Akinci F, Sinay T (2003). Perceived access in a managed care environment: determinants of satisfaction. J Health Manag Res.

[25] Quintana JM, González N, Bilbao A, Aizpuru F, Escobar A, Esteban C (2006). Predictors of patient satisfaction with hospital health care. BMC Health Serv Res.

[26] Iran CotIRo. Islamic Republic of Iran. Archived from the original (Excel) 2011. 2011. p. 11.

[27] . Ghods A ME, Vanaki Z. Designing and psychometric testing of patient’s satisfaction questionnaire with nursing care Tehran, Iran: Tarbiat Modares University 2011.

[28] Khamis K, Njau B (2014). Patients’ level of satisfaction on quality of health care at Mwananyamala hospital in Dar es Salaam, Tanzania. BMC Health Serv Res.

[29] Karimi S kM (2008). Study of barriers to providing health services in the city of Sirjan between the years 2001- 2005. J Health Inf Manag.

[30] Son I-S, Hwang J-I (2007). The relationship between patient characteristics and satisfaction with hospital care. J Korean Acad Nurs.

[31] Findik UY, Unsar S, Sut N (2010). Patient satisfaction with nursing care and its relationship with patient characteristics. Nurs Health Sci.

[32] Peterson WE, Charles C, DiCenso A, Sword W (2005). The Newcastle Satisfaction with Nursing Scales: a valid measure of maternal satisfaction with inpatient postpartum nursing care. J Adv Nurs.

[33] Mogadasiyan S, Firoziyan A, Nikanafar A, Rahmani A, Abdolahzadeh F (2013). Satisfaction with nursing care and related factors in hospitalized cancer patients in shahid ghazi hospital in tabriz. The Journal of Urmia Nursing and Midwifery Faculty.

[34] Sweeney J, Brooks AM, Leahy A (2003). Development of the Irish National patient perception of quality of care survey. Int J Qual Health Care.

[35] Otani K, Herrmann PA, Kurz RS (2011). Improving patient satisfaction in hospital care settings. Health Serv Manag Res.

[36] Morris G, District O-E. Improving quality of services. Health Systems Trust, South African Health Review, Durban: The Press Gang. 1999.

[37] Al Doghaither A. Inpatient satisfaction with physician services at King Khalid University Hospital, Riyadh, Saudi Arabia. 2004. 16212213

[38] Hansen PM, Peters DH, Viswanathan K, Rao KD, Mashkoor A, Burnham G (2008). Client perceptions of the quality of primary care services in Afghanistan. Int J Qual Health Care.

[39] Gurung T. Factors in fluencing patient satisfaction in a free health care system in the National Referral Hospital (NRH), Thimphu, Bhutan: Bangkok: College of Public Health; 2003.

[40] Senarath U, Fernando DN, Rodrigo I (2006). Factors determining client satisfaction with hospital-based perinatal care in Sri Lanka. Trop Med Int Health.

[41] Ohara Y (2010). Communication between health care professionals and patients. Rinsho Byori.

[42] Xiao H, Barber JP (2008). The effect of perceived health status on patient satisfaction. Value in Health.

[43] Woodside AG, Frey LL, Daly RT (1989). Linking sort/ice anlity, customer satisfaction, and behavioral intention. J Health Care Mark.

[44] Margolis SA, Al-Marzouqi S, Revel T, Reed RL (2003). Patient satisfaction with primary health care services in the United Arab Emirates. Int J Qual Health Care.

[45] Becker G, Newsom E (2003). Socioeconomic status and dissatisfaction with health care among chronically ill African Americans. Am J Public Health.

[46] Seyf RM, Shahidzadeh MA (2006). Patient satisfaction: A study of Hamedan teaching and general hospitals. Payesh.

[47] Hajian K (2007). Satisfaction of hospitalized patients of health care services in Shahid Beheshti and Yahyanezhad hospitals of Babol. Journal of Babol University of Medical Sciences.

[48] Cho SH (2005). Inpatient satisfaction and dissatisfaction in relation to socio-demographics and utilization characteristics. J Korean Acad Nurs.

[49] Zewdie B, Tsion A, Mirkuzie W, Sudhakar M (2010). Determinants of satisfaction with health care provider interactions at health centres in central Ethiopia: a cross sectional study. BMC Health Serv Res.

[50] Husted H, Holm G, Jacobsen S (2008). Predictors of length of stay and patient satisfaction after hip and knee replacement surgery: fast-track experience in 712 patients. Acta Orthop.

[51] Fan VS, Burman M, McDonell MB, Fihn SD (2005). Continuity of care and other determinants of patient satisfaction with primary care. J Gen Intern Med.

[52] Schoenfelder T, Klewer J, Kugler J (2011 Jun 29). Determinants of patient satisfaction: a study among 39 hospitals in an in-patient setting in Germany. Int J Qual Health Care.

[53] Jenkinson C, Coulter A, Bruster S, Richards N, Chandola T (2002). Patients’ experiences and satisfaction with health care: results of a questionnaire study of specific aspects of care. Qual Saf Health Care.

[54] Al-Windi A (2005). Predictors of satisfaction with health care: A primary healthcare-based study. Qual Prim Care.

